# Genomic Insights into Carbapenem-Resistant Organisms Producing New Delhi Metallo-β-Lactamase in Live Poultry Markets

**DOI:** 10.3390/microorganisms13061195

**Published:** 2025-05-23

**Authors:** Xueqiang Xin, Yi Yin, Jiayong Kong, Mianzhi Wang, Zhiqiang Wang, Ruichao Li

**Affiliations:** 1 Jiangsu Co-Innovation Center for Prevention and Control of Important Animal Infectious Diseases and Zoonoses, College of Veterinary Medicine, Yangzhou University, Yangzhou 225009, China; 2 Institute of Comparative Medicine, Yangzhou University, Yangzhou 225009, China; 3 Joint International Research Laboratory of Agriculture and Agri-Product Safety, the Ministry of Education of China, Yangzhou University, Yangzhou 225009, China

**Keywords:** live poultry, carbapenem resistance, *bla*_NDM_, genomic analysis, transmission

## Abstract

New Delhi metallo-β-lactamase (NDM) is an enzyme that can degrade a wide range of β-lactam antibiotics. The widespread dissemination of the *bla*_NDM_ gene, which encodes NDM, in animal-derived settings poses a threat to public health security. Live poultry markets represent critical nodes in public health surveillance. However, there is currently limited reporting on the spread of the *bla*_NDM_ gene within these markets under the One Health approach. This study investigated the prevalence of the *bla*_NDM_ gene in live poultry markets and, by integrating newly sequenced genomes with publicly available database entries, performed an in-depth analysis of its association networks with other genetic elements across species. A total of 233 *bla*_NDM_-positive strains, comprising 218 *Escherichia coli* strains, 4 *Enterobacter cloacae* strains, 7 *Klebsiella pneumoniae*, 2 *Klebsiella aerogenes*, 1 *Providencia rettgeri*, and 1 *Proteus mirabilis* were isolated from two live poultry markets in Jiangsu, China. Among the *bla*_NDM_-positive strains, multiple variants were identified, primarily *bla*_NDM-5_, followed by *bla*_NDM-1_, *bla*_NDM-13_, *bla*_NDM-27_, and *bla*_NDM-39_. The coexistence of *bla*_NDM-5_ and *mcr-1* was detected in five *E. coli* strains. Additionally, we found one *E. coli* strain in which *bla*_NDM-5_ coexisted with *estT* and *tet*(X4), and another *E. coli* strain where *bla*_NDM-5_ coexisted with *estT*. Spearman correlation analysis of publicly available genomes revealed that the genetic element preferences of *bla*_NDM_ variants vary significantly across species (|R| > 0.3, *p* < 0.05). The element preferences of *E. coli* strains carrying *bla*_NDM-5_ are similar to those of *Klebsiella pneumoniae* harboring *bla*_NDM-1_. In *Klebsiella aerogenes*, *Enterobacter cloacae*, and *Proteus mirabilis*, strains carrying *bla*_NDM-1_, have opposite genetic element preferences when compared with strains harboring *bla*_NDM-5_ or *bla*_NDM-7_. Notably, we report the first evidence of the *bla*_NDM-1_ gene transfer mediated by IS*Kpn13*, IS*Spu2*, and MITE*Kpn1*. The findings highlight that live poultry markets are important transmission hotspots of AMR and thus require continuous surveillance.

## 1. Introduction

Antibiotics serve as the principal therapeutic agents employed by humans in the battle against a wide spectrum of infectious diseases and have exerted substantial positive impacts on human and animal health within the domains of medicine, animal husbandry, and food safety. However, with the widespread use of antibiotics, antimicrobial resistance (AMR) has become a major threat to global public health, and the increasing multidrug resistance (MDR) in clinical pathogens has further exacerbated the problem. Horizontal gene transfer of antibiotic resistance genes (ARGs) across ecological niches amplifies the risk of clinical resistance. Globally, live poultry markets are high-risk interfaces for human–animal contact. These markets aggregate poultry from diverse regions, facilitating ARG transfer and pathogen dissemination [1]. Live poultry markets have been proven to be reservoirs and dissemination centers for ARGs [2]. The ARGs detected in people, poultry, and the environment within the markets are more diverse than those detected in poultry farms [3]. This indicates that the risk of ARGs spreading through food animals is high, and that they can easily be further disseminated through pathways such as water and air [4], posing a threat to the entire public health security.

Carbapenems, a class of atypical β-lactam antibiotics, are broad-spectrum agents reserved for treating multidrug-resistant (MDR) infections in human medicine and are prohibited for use in veterinary practice [5]. They serve as a last-line defense in clinical settings. Nevertheless, the increasing prevalence of carbapenem-resistant gram-negative bacteria in recent years has raised significant concerns in the global public health community. The *bla*_NDM_ gene, which was first identified in 2009 and encodes New Delhi metallo-β-lactamase (NDM), is a clinically significant determinant of carbapenem resistance [6]. As the product of the *bla*_NDM_ gene, NDM enzymes can degrade the majority of β-lactam antibiotics, including carbapenems such as meropenem and imipenem [6], thereby compromising the effectiveness of these agents against pathogens that harbor the *bla*_NDM_ gene. The *bla*_NDM_ gene is predominantly found within the Gammaproteobacteria, specifically in families such as *Enterobacteriaceae*, *Moraxellaceae*, *Morganellaceae*, and *Pseudomonadaceae* [7], and it does not spread across different classes [8]. Additionally, the majority of *bla*_NDM_ gene variants have been identified in *Enterobacteriaceae* bacteria. Within this family, *bla*_NDM_-positive strains of *Klebsiella pneumoniae*, *Escherichia coli*, and *Enterobacter cloacae* are the most numerous and widely spread [7]. Therefore, to curb the spread of the *bla*_NDM_ gene, it is crucial to implement the global surveillance of *bla*_NDM_-positive *Enterobacteriaceae* bacteria under the One Health framework. To date, the spread of the *bla*_NDM_ gene across different ecological niches has been extensively documented [9,10,11,12,13]. However, information regarding the prevalence of *bla*_NDM_ in live poultry markets remains limited.

In this study, 351 meropenem-resistant strains were isolated from 388 non-duplicate samples and 233 *bla*_NDM_-positive strains were identified from various ecological niches within live poultry markets. Through whole-genome sequencing (WGS), we elucidated the genomic characteristics of these *bla*_NDM_-harboring strains. By integrating the sequenced genomes with those available in databases, we conducted a comprehensive analysis of the association networks between various *bla*_NDM_ gene variants and other genetic elements across different species.

## 2. Materials and Methods

### 2.1. Sample Collection and Strain Identification

In July 2022, a total of 388 non-duplicate samples were collected from two large-scale live poultry markets in Yangzhou to investigate the epidemiology of *bla*_NDM_-positive strains in both animals and the environment. The poultry traded in these markets originated from Anhui Province and several cities in Jiangsu Province, including Huai’an, Nanjing, Nantong, Taizhou, Yangzhou, and Yancheng. The samples comprised animal feces (chicken, *n* = 159; duck, *n* = 29; goose, *n* = 66; pigeon, *n* = 21) and other samples (soil, *n* = 17; water, *n* = 36; environment, *n* = 57; plant, *n* = 3) (Table S1). All samples were transported to the laboratory in cool boxes with ice packs (4 °C) for bacterial cultivation and DNA extraction. The collected samples were transferred into 2 mL brain heart infusion (BHI) liquid broth (Haibo, Qingdao, China) and incubated at 37 °C for 6 h for pre-bacterial growth. Preculture samples were then spread onto MacConkey (Haibo, Qingdao, China) plates supplemented with 2 mg/L meropenem (Aladdin, Shanghai, China) and incubated for 18 h at 37 °C. Different colored colonies were selected from each plate to identify carbapenem-resistant isolates. All confirmed carbapenem-resistant strains were tested for the presence of *bla*_NDM_ genes via PCR, using the primers listed in Table S2. All *bla*_NDM_-positive bacteria were identified using MALDI-TOF MS Axima^TM^ (Shimadzu, Nakagyo-ku, Japan) and 16S rRNA gene sequencing (Table S2).

### 2.2. Antimicrobial Susceptibility Testing

Antimicrobial susceptibility was tested using the broth dilution method [14]. The susceptibility of carbapenem-resistant isolates was evaluated for a range of antimicrobial drugs (all purchased from Aladdin, Shanghai, China) commonly used in both human medicine and veterinary practice, including meropenem (MEM), imipenem (IMP), ampicillin (AMP), ceftazidime (CAZ), kanamycin (KAN), gentamicin (GEN), ciprofloxacin (CIP) and colistin (CL), in accordance with the recommendations of the Clinical and Laboratory Standards Institute (CLSI) for antimicrobial susceptibility testing of *Enterobacterales* [14]. Minimum inhibitory concentrations (MICs) were interpreted in accordance with the guidelines provided by the CLSI (2021) [14] and the breakpoint tables specified in the European Committee on Antimicrobial Susceptibility Testing v.12.0 [15]. *E. coli* ATCC 25,922 was used as a quality control strain.

### 2.3. Plasmid Conjugation Assay

To explore the transferability of genetic elements carrying the *bla*_NDM_ gene, we conducted a conjugation assay using rifampicin-resistant *E. coli* C600 as the recipient strain. The liquid mating method was utilized for this purpose [16]. Initially, overnight cultures of the original isolates and recipient strains were prepared in Luria–Bertani (LB) liquid broth (Haibo, Qingdao, China). These cultures were subsequently adjusted to an optical density of 0.6 at 600 nm. A volume of 50 μL of the mixed bacterial cultures was then pipetted and evenly spread onto LB solid media containing 100 μg/mL rifampicin (Aladdin, Shanghai, China) and 2.0 μg/mL meropenem. Following an overnight incubation at 37 °C, single bacterial colonies were selected for PCR analysis to confirm the successful transfer of the *bla*_NDM_ gene.

### 2.4. Whole-Genome Sequencing of bla_NDM_-Positive Strains

Based on the bacterial species and the MIC results, 38 *bla*_NDM_-positive strains were selected for whole-genome sequencing. It should be noted that, among the 38 *bla*_NDM_-positive strains sequenced, six strains were derived from the previous study [17] and represent extensions of prior research. The genomes of 38 *bla*_NDM_-positive strains were extracted using the FastPure Bacteria DNA Isolation Mini Kit (Vazyme, Nanjing, China). The concentration and purity of the extracted DNA were evaluated using NanoDrop 2000 (Thermofisher, Waltham, MA, USA) and gel electrophoresis, with the final concentration precisely determined using the Qubit^TM^ 4.0 fluorometer (Invitrogen, Carlsbad, CA, USA). Subsequently, short-read sequencing was performed on the extracted DNA using DNBseq (BGI, Shenzhen, China), producing paired-end reads of 2 × 150 bp. The collected raw reads, with a minimum coverage of 100 fold, were then processed for trimming using SOAPnuke v.2.17 [18]. De novo assembly of clean reads was subsequently carried out using SPAdes v.3.13.1 [19].

### 2.5. Bioinformatics Analysis of Assembled Genomes

Mlst v.2.23.0 (https://github.com/tseemann/mlst, accessed on 10 August 2023) was used to determine the multi-locus sequence type (MLST) of all assembled genomes. Resfinder [20], ISfinder [21], Plasmidfinder [22], VFDB core dataset [23] and ICEberg [24] were run with 80% coverage and 80% identity in Abricate (https://github.com/tseemann/abricate, accessed on 7 July 2020) to identify ARGs, insertion sequences (ISs), plasmid replicons, and integrating conjugative elements (ICEs). ECTyper [25] was used to identify serotypes of all *E. coli* genomes. PHASTER [26] was employed to identify prophage sequences, and PlasmidHunter [27] was utilized to detect plasmid-like contigs. Prokka v.1.14.6 [28] was used to conduct genome annotation. Phylogenetic trees were constructed using Roary v.3.13.0 [29] and FastTree v.2.1.11 [30] and visualized using Chiplot (https://www.chiplot.online, accessed on 20 March 2025). Heatmap was drawn using Chiplot. The genetic environment of plasmids was visualized using BRIG v.0.95 [31].

### 2.6. Genetic Environment Analysis of bla_NDM_-Positive Strains

In order to analyze the differences in the genetic environment among different *bla*_NDM_ gene variants, Abricate (https://github.com/tseemann/abricate, accessed on 10 August 2023) was used to identify 4072 *bla*_NDM_-positive strains (Table S3) from the Carbapenem-resistant *Escherichia coli* (CREC) dataset of a previous study [32]. We also downloaded 66,609 genomes from *Klebsiella* genus, 10,762 genomes from *Enterobacter* genus and 3446 genomes from *Proteus* genus from the NCBI database Release 258.0 (as of 10 December 2023). CheckM2 v.1.0.2 [33] was used to identify genomes with over 95% completeness and less than 5% contamination. Feature information, including collection date, host, country, species and isolation source of these genomes was collected using a homemade python script. Linear genomic comparison and bar plot were visualized using ChiPlot. The network graph depicting the coexistence patterns of different *bla*_NDM_ gene variants with other ARGs, ISs and plasmid replicons was constructed using Gephi v.0.10.1 [34].

### 2.7. Identification of bla_NDM_-Positive Pathogenic E. coli Strains

To identify the *bla*_NDM_-positive pathogenic *E. coli* strains, according to the previous study [35], we classified strains harboring either the *elt* or *est* gene as Enterotoxigenic *E. coli* (ETEC), and those carrying either the *aatA* or *aaiC* gene as Enteroaggregative *E. coli* (EAEC). Strains containing the *eae* gene, but which were negative for the *bfpA* gene, were identified as atypical Enteropathogenic *E. coli* (EPEC), while strains carrying the *stx* and *eae* genes were categorized as Enterohemorrhagic *E. coli* (EHEC). A total of 4072 *bla*_NDM_-positive CREC strains and 29 *E. coli* strains isolated in this study were identified with the aforementioned virulence genes.

### 2.8. Statistical Analysis

Statistical analysis and plotting were performed using R v.4.3.1 (R Foundation for Statistical Computing, Vienna, Austria). Spearman correlation analysis was used to determine the correlation among the *bla*_NDM_ gene, other ARGs, ISs and plasmid replicons. Variable pairs with an absolute Spearman correlation coefficient (R) greater than 0.3 were considered to have a strong correlation (|R| > 0.3). Only variable pairs with a *p*-value less than 0.05 were included in the analysis.

## 3. Results

### 3.1. bla_NDM_-Positive Strains Profile

A total of 388 original samples were collected from two live poultry markets in Yangzhou, China. A total of 351 meropenem-resistant strains were isolated from these samples, among which 233 strains were *bla*_NDM_-positive (isolated from 144 original samples). The *bla*_NDM_ detection rates were 37.11% (144/388) among samples and 66.38% (233/351) among meropenem-resistant isolates. Among the 233 *bla*_NDM_-positive isolates, 130 were derived from 71 unique samples collected at live poultry market A, resulting in a positive rate of 31.42% (71/226) at this market. The remaining 103 isolates were obtained from 73 unique samples at live poultry market B, with a positive rate of 45.06% (73/162) (Table 1).

Among the 233 *bla*_NDM_-positive strains, there were 218 *Escherichia coli* strains (93.56%), 4 *Enterobacter cloacae* strains (1.72%), 7 *Klebsiella pneumoniae* (3.00%), 2 *Klebsiella aerogenes* (0.86%), 1 *Providencia rettgeri* (0.43%), and 1 *Proteus mirabilis* (0.43%). Conjugation assays were conducted on the 233 *bla*_NDM_-positive strains, and ultimately 91 *E. coli* C600 transconjugants were obtained, with a conjugation success rate of 39.10%. Among the strains that successfully transferred the *bla*_NDM_ gene through conjugation, all were *E. coli* except for 3 *E. cloacae* strains and 1 *Proteus mirabilis* strain.

A total of 233 *bla*_NDM_-positive strains were tested for susceptibility to a variety of antibiotics (Table S14). The tested strains exhibited extremely high resistance to meropenem, imipenem, ampicillin, and ceftazidime, with resistance rates approaching 100%. Among the aminoglycoside antibiotics, resistance rates to kanamycin and gentamicin were also high, reaching 87.12% and 82.40%, respectively. Additionally, the tested strains showed a resistance rate of 74.25% to ciprofloxacin and 20.12% to colistin. Only 11.19% (15/134) of strains isolated from chickens were resistant to colistin, while 50% (17/34) of strains from environmental sources were resistant to colistin. Although colistin demonstrated relatively good antimicrobial activity against *bla*_NDM_-positive strains, the presence of resistance must be taken seriously and monitored more closely.

### 3.2. Genomic Analysis of bla_NDM_-Positive Strains

To investigate the genetic characteristics of *bla*_NDM_-positive strains, 38 representative *bla*_NDM_-positive strains were selected for whole-genome sequencing and analysis, including 29 *E. coli* strains, 4 *E. cloacae* strains, 2 *K. pneumoniae* strains, 2 *K. aerogenes* strains, and 1 *P. mirabilis* strain. For the 29 *E. coli* strains, the average genome size was 5.16 Mb with an average GC content of 50.34%. Table S4 summarizes the genomic information of all sequenced strains. Additionally, the length range of contigs carrying *bla*_NDM_ gene generated by DNBseq sequencing is 3315 to 45,493 bp.

**Figure 1:**
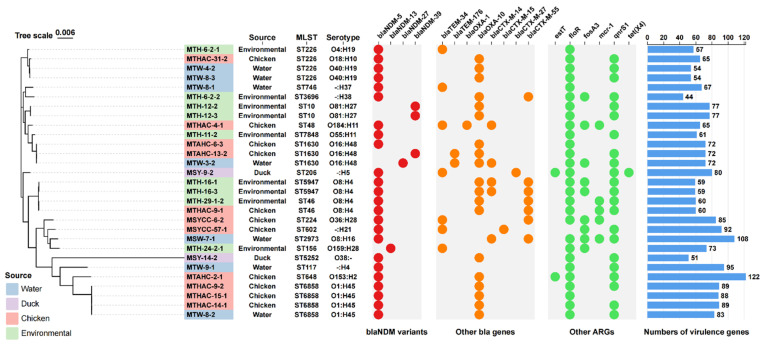
The phylogenetic tree and ARG heatmap of 29 *E. coli* isolates. The phylogenetic tree was generated by FastTree based on core gene alignment using Roary and was visualized using Chiplot. Isolates from different sources are highlighted in different colors. The three columns of information marked next to the strain names are isolation source, ST type (identified by MLST), and serotype (identified by ECTyper). The three sets of heatmaps show the presence of ARGs in the strains. The outermost bar chart shows the number of virulence genes in the isolates based on the VFDB core dataset.

Based on core genome SNPs, we constructed a phylogenetic tree of 29 *bla*_NDM_-positive *E. coli* strains (Figure 1). The 29 *E. coli* strains from this study presented 18 distinct sequence types, with ST226 (13.79%, 4/29), ST6858 (13.79%, 4/29) and ST1630 (10.34%, 3/29) being the most prominent. A total of 19 serotypes were identified, mainly including O1:H45 (13.79%, 4/29), O8:H4 (13.79%, 4/29), and O16:H48 (10.34%, 3/29). The number of virulence genes of all the *E. coli* strains were counted based on the VFDB core datasets. It is worth noting that one strain of serotype O153:H2 *E. coli* carries 122 virulence genes, and one strain of serotype O8:H16 *E. coli* carries 108 virulence genes (Figure 1). Further investigation revealed that, in the O153:H2 *E. coli* strain, a 13.8 Kb plasmid-like contig harbored the *iro* genes, while a 12.3 Kb plasmid-like contig carried the *iuc* genes. Similarly, in the O8:H16 *E. coli* strain, a 19.9 Kb plasmid-like contig was found to contain the *iuc* genes. During this process, we observed that, among all of the sequenced *E. coli* strains, a total of five strains carried the *iuc* gene cluster, and these strains also harbored a substantial number of virulence genes (100 ± 15). By identifying specific virulence genes in the 29 *E. coli* strains isolated in this study and the 4072 *bla*_NDM_-positive CREC strains collected, we further discovered that the characteristic virulence genes of pathogenic *E. coli* were not identified in the sequenced strains. However, among the 4072 *bla*_NDM_-positive CREC strains, 7 atypical EPEC strains and 23 EAEC strains (17 strains harbored only the *aatA* gene, 5 strains harbored only the *aaiC* gene, and 1 strain carried both genes) were identified. No ETEC or EHEC strains were detected.

Among the 29 *bla*_NDM_-positive *E. coli* strains, 24 harbored the *bla*_NDM-5_ gene. The remaining strains included three with *bla*_NDM-39_, one with *bla*_NDM-13_, and one with *bla*_NDM-27_. Co-occurring β-lactamase genes included *bla*_OXA-10_ (19/29, 65.52%), with multiple *bla*_TEM_ and *bla*_CTX-M_ variants also present. Moreover, the *floR* gene was carried by almost all strains (96.55%, 28/29), and the majority of strains also harbored the *qnrS1* gene (75.86%, 22/29). As a result, most of these *E. coli* strains were resistant to fluoroquinolone antibiotics. However, we observed an intriguing phenomenon among the four ST6858, O1:H45 *E. coli* strains that are closely related in terms of evolutionary relationships (Figure 1). Three of these strains carried both the *floR* and *qnrS1* genes, while one strain carried only the *floR* gene. However, the MIC results (Table S14) show that two strains (including the one carrying only *floR*) are highly resistant to the fluoroquinolone antibiotic ciprofloxacin (MIC > 128), while the other two strains are susceptible to ciprofloxacin (MIC = 0.5). Additionally, no mutations in the *gyrA* and *parC* genes were detected in these four strains. This discrepancy may suggest the presence of undiscovered genetic mutations or regulatory effects. It is noteworthy that the coexistence of the colistin resistance gene *mcr-1* and *bla*_NDM-5_ was found in five strains, and that the coexistence of the tigecycline resistance gene *tet*(X4) and *bla*_NDM-5_ was detected in one strain. Furthermore, the resistance gene *estT* encoding macrolide hydrolase was identified in two strains (Figure 1).

ARGs harbored by *E. cloacae*, *K. pneumoniae*, *K. aerogenes*, and *P. mirabilis* differed from that harbored by *E. coli* (Figure S1). Except for *P. mirabilis*, which harbored *bla*_NDM-1_, all other strains carry *bla*_NDM-5_. Additionally, *bla*_OXA-10_ was detected in two *K. aerogenes* strains and one *P. mirabilis* strain. Moreover, *bla*_TEM-176_ and *bla*_TEM-1B_ were identified in two *K. pneumoniae* strains. Except for two *E. cloacae* strains, all other strains harbored the *floR* gene. Furthermore, strains from different genera carried different variants of the *fosA* gene: *E. cloacae* carried *fosA2*, *P. mirabilis* carried *fosA3*, *K. aerogenes* carried *fosA5* and *fosA7*, and *K. pneumoniae* carried *fosA6*.

### 3.3. Genetic Environment Analysis of Various bla_NDM_ Gene Variants

**Figure 2:**
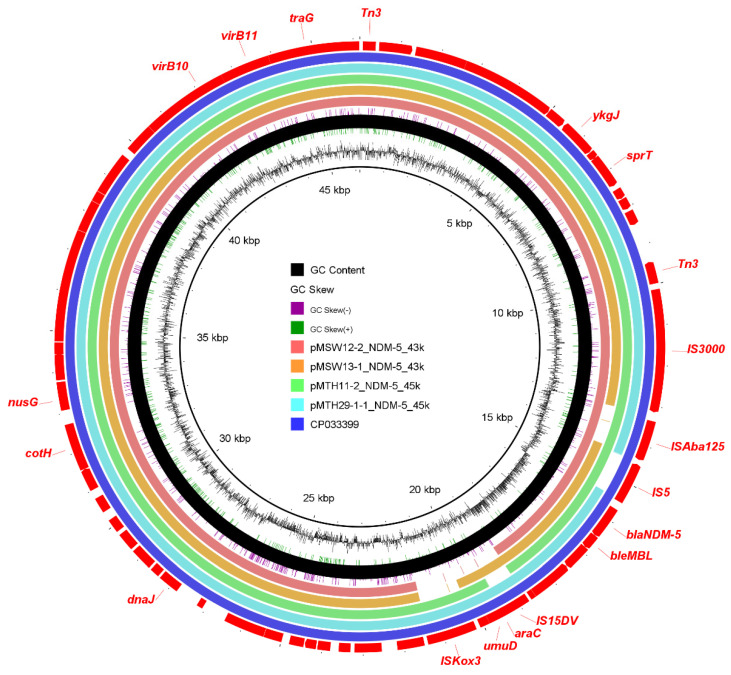
Plasmid profile of the *bla*_NDM-5_-containing IncX3-type plasmid. Plasmid slices (assembled contigs, not complete plasmids) from this study were compared with a plasmid (CP033399.1) derived from *E. coli*. The GC skew and GC content are depicted in an inward-to-outward sequence. The outermost arrows colored in red indicate the positions and transcriptional orientations of the open reading frames.

Multiple plasmid replicon types were detected in all of the *bla*_NDM_-positive strains, but we only observed that the *bla*_NDM-5_ gene is directly located on the IncX3-type plasmids in three *E. cloacae* and one *E. coli* (Figure 2). The transfer of *bla*_NDM-5_ was mediated by the upstream IS*5* or IS*Aba125*. In addition, in two strains of *E. coli*, the *mcr-1* gene was respectively located on a 60 kb Incl2-type plasmid and a 105 kb IncHI2A-type plasmid (Figure S2).

**Figure 3:**
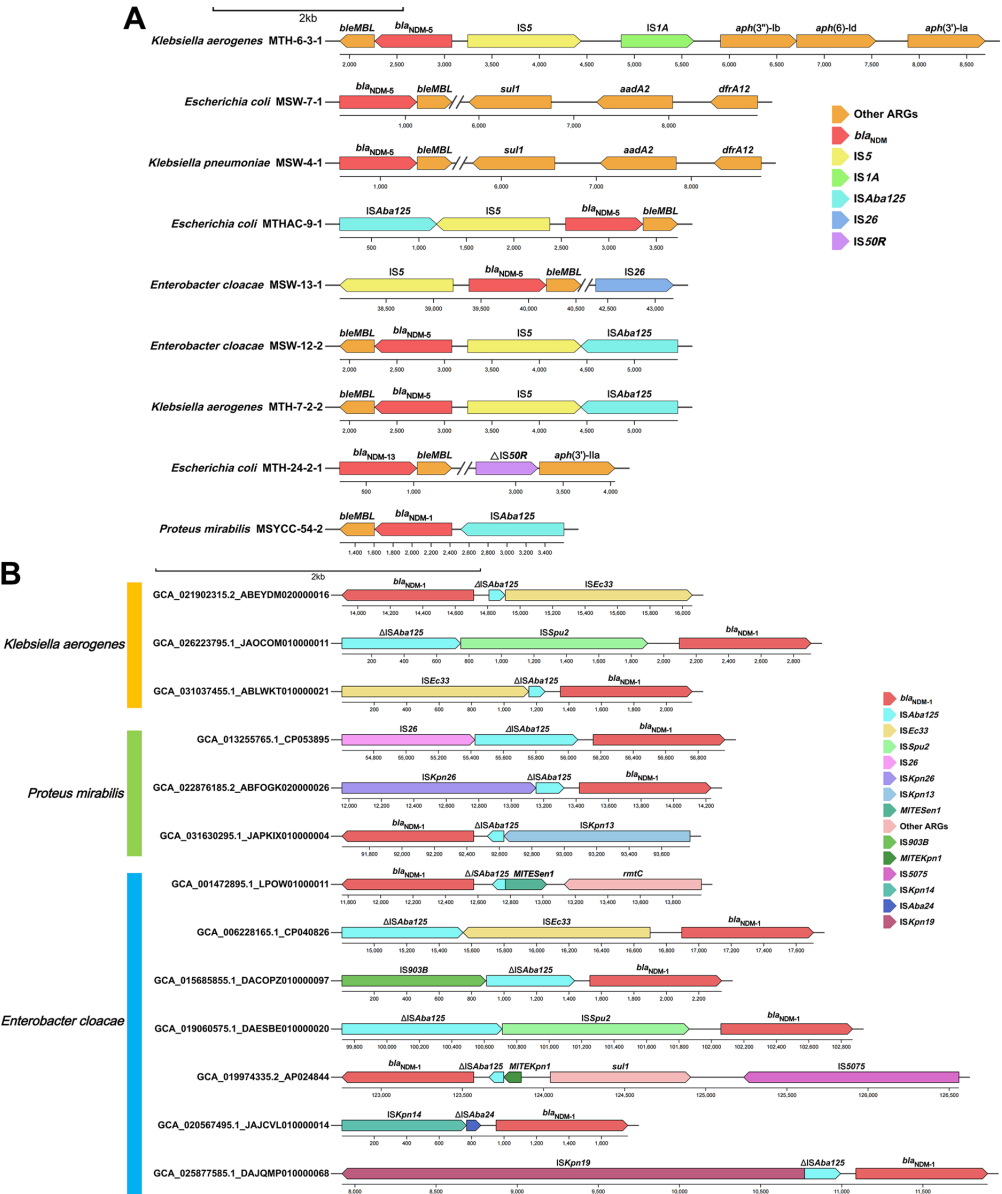
Genetic environments of several *bla*_NDM_ variants in assembled genomes and genomes from the database. (**A**) Different types of genetic environments of *bla*_NDM_ variants in assembled genomes. Different genetic elements are highlighted in different colors. (**B**) Primary genetic contexts of *bla*_NDM-1_ across different species. In different species, the transfer of *bla*_NDM-1_ is mediated by various IS elements.

Genetic environment analysis revealed the diversity of *bla*_NDM_ variants bearing genetic contexts. IS*Aba125*-IS*5*-*bla*_NDM-5_-*ble*_MBL_ was the most common transposable structure found in *E. coli*, *E. cloacae*, and *K. aerogenes* (Figure 3A). In another *K. aerogenes*, the genetic structure of *ble*_MBL_-*bla*_NDM-5_-IS*5*-IS*1A*-*aph*(3″)-Ib-*aph*(6)-Id-*aph*(3″)-Ia was discovered. This genetic structure may have been formed by the insertion of IS*1A*-*aph*(3″)-Ib-*aph*(6)-Id-*aph*(3″)-Ia mediated by IS*1A*, which replaced the previous IS*Aba125*. Additionally, the IS*Aba125*-*bla*_NDM-1_-*ble*_MBL_ transposon structure was identified in one *P. mirabilis* strain.

### 3.4. Correlation Analysis of bla_NDM_ with Other ARGs, ISs and Plasmid Replicons

To thoroughly investigate the genetic background of the bla_NDM_ gene, we collected the CREC samples used in the previous study [32] and downloaded all of the genomes of the genera *Klebsiella*, *Enterobacter*, and *Proteus* from the NCBI database. Through sequence alignment, a total of 4072 *bla*_NDM_-positive CREC strains (Table S3), 8465 bla_NDM_-positive *K. pneumoniae* strains (Table S5), 84 *bla*_NDM_-positive *K. aerogenes* strains (Table S6), 139 *bla*_NDM_-positive *P. mirabilis* strains (Table S7), and 105 *bla*_NDM_-positive *E. cloacae* strains (Table S8) were identified.

Distinct distributions of *bla*_NDM_ variants were observed across species (Figure S3A). Upon analysis of the assembled genomes from this study and downloaded genomes, it was observed that 76.88% (3153/4101) of *bla*_NDM_-positive CREC strains harbored the *bla*_NDM-5_ gene, 15.51% (636/4101) possessed the *bla*_NDM-1_ gene, 3.71% (152/4101) carried the *bla*_NDM-7_ gene, and 1.95% (80/4101) contained the *bla*_NDM-4_ gene (Table S9). Notably, two CREC strains were found to concurrently harbor *bla*_NDM-1_, *bla*_NDM-4_, *bla*_NDM-5_, and *bla*_NDM-24_. In *K. pneumoniae* strains, the distribution was as follows: 66.78% (5653/8477) carried the *bla*_NDM-1_ gene, 27.23% (2305/8477) possessed the *bla*_NDM-5_ gene, 3.61% (306/8477) harbored the *bla*_NDM-7_ gene, and 1.44% (122/8477) contained the *bla*_NDM-4_ gene (Table S10). For *K. aerogenes* strains, the proportions were 44.18% (38/86) for the *bla*_NDM-1_ gene, 31.40% (27/86) for the *bla*_NDM-5_ gene, and 23.26% (20/86) for the *bla*_NDM-7_ gene (Table S11). In *bla*_NDM_-positive *P. mirabilis* strains, 70.71% (99/140) carried the *bla*_NDM-1_ gene, 22.86% (32/140) possessed the *bla*_NDM-7_ gene, and 6.43% (9/140) harbored the *bla*_NDM-5_ gene (Table S12). As for *bla*_NDM_-positive *E. cloacae* strains, 75.23% (82/109) carried the *bla*_NDM-1_ gene, while 20.18% (22/109) possessed the *bla*_NDM-5_ gene (Table S13).

Network graph analysis revealed that different *bla*_NDM_ gene variants in different species exhibit distinct preferences for genetic elements (Figures S3B,C and S4). When the absolute value of R is greater than 0.3 and *p* is less than 0.05, we consider that there is a correlation between different genetic elements. In CREC strains, *bla*_NDM-5_ was strongly correlated with *bla*_TEM-1B_, *bla*_CTX-M-15_, and *bla*_OXA-1_, while *bla*_NDM-1_ was strongly correlated with *bla*_SHV-12_ (R > 0.3, *p* < 0.05). In addition, *bla*_NDM-5_ was strongly correlated with ARGs such as *sul1*, *aadA2*, *mph(A)*, and insertion sequence IS*6100*, whereas *bla*_NDM-1_ was strongly correlated with *rmtC* and *aph*(3′)-VI, and *bla*_NDM-7_ was strongly correlated with IS*Cfr27* (R > 0.3, *p* < 0.05). However, unlike CREC strains, in *K. pneumoniae* strains, *bla*_NDM-5_ only showed positive associations with ARGs such as *rmtB*, *erm*(B), *oqxA*, *oqxB*, and *mph*(A), as well as the plasmid replicon IncX3, while *bla*_NDM-1_ was strongly correlated with *bla*_CTX-M-15_, *bla*_TEM-1B_, *bla*_OXA-1_, and *bla*_OXA-9_ (R > 0.3, *p* < 0.05). Additionally, *bla*_NDM-1_ was also strongly correlated with ARGs such as *oqxB*, *oqxA*, and *sul1* (R > 0.3, *p* < 0.05). In *K. aerogenes* strains, a distinct correlation pattern was observed. Genetic elements such as *bla*_SHV-12_, IS*Sen4*, IS*Cfr4*, and IS*Kpn26* were found to be strongly positively correlated with *bla*_NDM-1_ (R > 0.3, *p* < 0.05), while *floR*, IS*Aba125*, and IS*5* exhibited negative correlations with *bla*_NDM-1_ (R < −0.3, *p* < 0.05). Notably, plasmid replicons, including IncN2, IncHI1B, and IncFIB, were identified as being strongly positively correlated with *bla*_NDM-1_ (R > 0.3, *p* < 0.05), whereas IncX3 showed a negative correlation with *bla*_NDM-1_ (R < −0.3, *p* < 0.05). However, IncX3 and IS*5* were positively correlated with *bla*_NDM-5_ (R > 0.3, *p* < 0.05). Similar correlation patterns were also observed in *P. mirabilis* strains and *E. cloacae* strains. In both *P. mirabilis* strains and *E. cloacae* strains, *bla*_NDM-1_ was strongly negatively correlated with IS*Aba125*, IncX3, and IS*5* (R < −0.3, *p* < 0.05). In *P. mirabilis* strains, *bla*_NDM-7_ was positively correlated with IS*5* and IS*Aba125* (R > 0.3, *p* < 0.05); meanwhile, in *E. cloacae strains*, *bla*_NDM-5_ was positively correlated with IncX3, IS*5*, and IS*Aba125* (R > 0.3, *p* < 0.05). Additionally, in *P. mirabilis* strains, *bla*_NDM-1_ was positively correlated with *bla*_OXA-10_, *sul1*, *arr-3*, *aph*(3′)-Ia, and Col3M (R > 0.3, *p* < 0.05), and negatively correlated with IncC and *qnrS1* (R < −0.3, *p* < 0.05). In contrast, *bla*_NDM-7_ was positively correlated with *qnrS1*, IncC, and *floR* (R > 0.3, *p* < 0.05), and negatively correlated with Col3M, *sul1*, *arr-3*, and *aph*(3′)-Ia (R < −0.3, *p* < 0.05). In *E. cloacae* strains, *bla*_NDM-1_ was positively correlated with *bla*_CMH-3_ (R > 0.3, *p* < 0.05), and negatively correlated with IS*Kox3* and *floR* (R < −0.3, *p* < 0.05), while *bla*_NDM-5_ was positively correlated with IS*Kox3* and *floR* (R > 0.3, *p* < 0.05).

Unexpectedly, *bla*_NDM-1_ exhibited negative associations with IS*Aba125* and IS*5* in *K. aerogenes*, *P. mirabilis*, and *E. cloacae*—despite these IS elements being canonical mediators of *bla*_NDM-1_ transfer. We further investigated the genomic characteristics of *K. aerogenes* strains, *P. mirabilis* strains and *E. cloacae* strains and found that IS*Aba125* was often interrupted by various insertion sequences other than IS*5* (Figure 3B). In *K. aerogenes* strains, IS*Aba125* was interrupted by IS*Ec33* and IS*Spu2*. In *P. mirabilis* strains, IS*Aba125* was interrupted by IS*26*, IS*Kpn26*, and IS*Kpn13*. In *E. cloacae* strains, IS*Aba125* was interrupted by IS*Ec33*, IS*903B*, IS*Spu2*, MITE*Kpn1*, IS*Kpn14*, and IS*Kpn19*. This may suggest that different species capture the heterologous IS*Aba125*-*bla*_NDM-1_ transposon via different types of insertion sequences and integrate it into their own genomes to better adapt the *bla*_NDM-1_ gene to different genetic environments.

## 4. Discussion

Carbapenem-resistant *Enterobacteriaceae* of animal origin represent a critical group of antimicrobial-resistant pathogens. The increasing number of carbapenem-resistant isolates identified poses a severe threat to global public health security. The *bla*_NDM_ gene, which encodes NDM, is an important ARG associated with human clinical medicine. It was first identified in a clinical isolate of *K. pneumoniae* from a hospitalized patient [6]. Although it is only prevalent in Gammaproteobacteria [8], it has had a significant impact on human clinical medicine [36,37,38,39,40], markedly reducing the efficacy of clinical treatments.

Live poultry markets serve as reservoirs and dissemination centers for ARGs [2]. The convergence of live poultry from various regions significantly amplifies the risk of ARG spread. Given the close contact between humans, animals, and the environment in live poultry markets, establishing a “One Health” AMR monitoring system in these settings is crucial for preventing the transmission of multidrug-resistant pathogens and for devising effective containment strategies [41]. In this study, we investigated *bla*_NDM_ -positive strains in two live poultry markets in Jiangsu Province, China. Over 90% of identified *bla*_NDM_-positive strains were *E. coli*, indicating the widespread presence of CREC strains in poultry. This may be because the *bla*_NDM_-bearing plasmids have a high fitness cost in other *Enterobacteriaceae* bacteria, but there is still a risk of further dissemination. Additionally, conjugation assays revealed that nearly 40% of *bla*_NDM_-positive strains harbored transferable *bla*_NDM_ genes, suggesting that the *bla*_NDM_ gene can be widely disseminated in live poultry markets.

We obtained assembled genomes of 38 *bla*_NDM_-positive strains through whole-genome sequencing. In five *E. coli* isolates, the coexistence of *bla*_NDM-5_ and *mcr-1* was detected. This once again demonstrates that, despite China’s ban on the use of colistin as a growth promoter in animal husbandry, which did not prohibit its use in veterinary treatment, animal sources still harbor stable populations of *E. coli* that are resistant to both carbapenems and colistin [42]. We posit that the improper utilization of colistin by farms and small-scale farmers for the prevention and control of animal bacterial infections could be a contributing factor to this phenomenon. Additionally, the previous study indicates that the *mcr-1* gene is more frequently detected in clinical patients who have received carbapenem treatment [43]. This suggests that *E. coli* carrying the *mcr-1* gene may more readily acquire additional resistance genes, potentially explaining the coexistence of *bla*_NDM_ and *mcr-1* genes. It is particularly noteworthy that multidrug-resistant plasmids carrying both *bla*_NDM_ and *mcr-1* genes have been detected in CREC strains isolated from clinical patients [44]. This indicates that the co-dissemination of *bla*_NDM_ and *mcr-1* genes is still strengthening, in turn implying that the resistance issues of colistin and carbapenems remain very serious.

We identified the number of virulence genes in the sequenced strains and explored the pathogenicity typing of *bla*_NDM_-positive CREC strains. Notably, in one strain of O8:H16 serotype *E. coli* isolate coharboring *bla*_NDM-5_ and *mcr-1*, 108 virulence genes were identified, indicating the potential for the spread of highly pathogenic multidrug-resistant bacteria in live poultry markets. Moreover, 122 virulence genes were identified in an O153:H2, ST648-type *bla*_NDM-5_-positive *E. coli* strain. ST648-type *E. coli* is considered a high-risk, globally epidemic clone that can cause human infections [45]. These findings serve as a warning for the sanitation efforts in live poultry markets. In addition, although our study’s dataset did not identify any ETEC or EHEC strains, and only a limited number of EPEC and EAEC strains were detected, the pathogenicity of *bla*_NDM_-positive CREC warrants further investigation. In studies focusing on CREC strains from children with diarrhea in Ethiopia, agricultural matrices in South Africa, and drinking water in Jordan, *bla*_NDM_-positive O157:H7 CREC strains and ETEC strains were identified [46,47,48]. This highlights the ongoing need to guard against the spread of pathogenic *bla*_NDM_-positive CREC strains.

Genetic environment analysis of assembled genomes from this study revealed that the *bla*_NDM_ gene was commonly transferred via IS*Aba125* or IS*5*. However, surprisingly, through network analysis of downloaded *bla*_NDM_-positive strains from the database, we found that, in *K. aerogenes* strains, *P. mirabilis* strains, and *E. cloacae* strains, the *bla*_NDM-1_ gene was negatively correlated with IS*Aba125* and IS*5*, which is contrary to the common situation. Upon further investigation, we discovered that, in *bla*_NDM-1_-positive *K. aerogenes* strains, *P. mirabilis* strains, and *E. cloacae* strains that lack IS*Aba125* (actually harboring truncated sequences), different insertion sequences interrupt the IS*Aba125*. Among these insertion sequences, the transfer of the *bla*_NDM-1_ gene mediated by IS*Ec33* [49,50], IS*6100* [51], IS*903B* [52], IS*Kpn14* [53], IS*Kpn19* [54], IS*Kpn26* [52], and MITE*Sen1* [52] has been reported. However, to our knowledge, this study is the first to report the transfer of the *bla*_NDM-1_ gene mediated by IS*Kpn13*, IS*Spu2*, and MITE*Kpn1*. In addition, regarding the fact that IS*Aba125* is frequently truncated by various types of insertion sequences across different species, we hypothesize that this phenomenon may result from the adaptation of certain insertion sequences to the genomes of these species. This adaptation allows insertion sequences for the capture of the IS*Aba125*-*bla*_NDM-1_ transposon and its integration into the genetic environment of the respective strains.

## 5. Conclusions

Overall, these findings indicate that the prevalence of carbapenem-resistant strains in live poultry markets is a cause for concern. The potential spread of highly virulent, multidrug-resistant pathogens underscores the importance of comprehensive surveillance efforts. Moreover, the molecular mechanisms by which strains of different species capture the *bla*_NDM-1_ gene warrant further investigation. Herein, we call for enhanced sanitation management in live poultry markets, the implementation of appropriate measures to curb the dissemination of *bla*_NDM_-positive strains, and the safeguarding of food safety in animal husbandry through a One Health approach.

## Figures and Tables

**Table 1:** Prevalence of *bla*_NDM_-positive strains isolated from different source in two live poultry market.

Collection Location	Source Location	Sample Type	No. of Samples	No. of Positive Samples (%)
Live poultry market A	Anhui	Chicken manure	18	13 (72.22)
		Duck manure	13	4 (30.77)
		Pigeon manure	12	3 (25)
	Huai’an	Chicken manure	31	22 (70.97)
	Nanjing	Chicken manure	13	1 (7.69)
	Nantong	Chicken manure	6	3 (50)
	Taizhou	Chicken manure	4	2 (50)
	Yangzhou	Environment	33	15 (45.45)
		Goose droppings	64	0 (0)
		Plant	3	0 (0)
		Soil	9	0 (0)
		Water	20	8 (40)
Live poultry market B	Nantong	Chicken manure	30	11 (36.67)
	Yancheng	Chicken manure	57	29 (50.87)
	Yangzhou	Duck manure	16	12 (75)
		Environment	24	5 (20.83)
		Goose droppings	2	2 (100)
		Pigeon manure	9	3 (33.33)
		Soil	8	0 (0)
		Water	16	11 (68.75)

## Data Availability

WGS data generated from this study are openly available at the China National GeneBank Database (CNGBdb) with accession number of CNP0007032.

## Supplementary Materials

The following supporting information can be downloaded at https://www.mdpi.com/article/10.3390/microorganisms13061195/s1, Table S1: Sample collection information for live poultry markets; Table S2: Primers used in this study for PCR; Table S3: Feature information of *bla*_NDM_-positive CREC strains; Table S4: Feature information of assembled genomes carrying *bla*_NDM_ genes from this study; Table S5: Feature information of *bla*_NDM_-positive *Klebsiella pneumoniae* strains; Table S6: Feature information of *bla*_NDM_-positive *Klebsiella aerogenes* strains; Table S7: Feature information of *bla*_NDM_-positive *Proteus mirabilis* strains; Table S8: Feature information of *bla*_NDM_-positive *Enterobacter cloacae* strains; Table S9: The prevalence of various *bla*_NDM_ variants in *bla*_NDM_-positive CREC strains; Table S10: The prevalence of various *bla*_NDM_ variants in *bla*_NDM_-positive *Klebsiella pneumoniae* strains; Table S11: The prevalence of various *bla*_NDM_ variants in *bla*_NDM_-positive *Klebsiella aerogenes* strains; Table S12: The prevalence of various *bla*_NDM_ variants in *bla*_NDM_-positive *Proteus mirabilis* strains; Table S13: The prevalence of various *bla*_NDM_ variants in *bla*_NDM_-positive *Enterobacter cloacae* strains; Table S14: Antimicrobial susceptibility profiles of 233 *bla*_NDM_-positive strains; Figure S1: The prevalence of ARGs harbored by *E. cloacae*, *K. aerogenes*, *K. pneumoniae* and *P. mirabilis*; Figure S2: Plasmid profiles of two mcr-1-containing plasmids. The annotation of two plasmid slices (assembled contigs, not complete plasmids) from this study. The GC skew and GC content are depicted in an inward-to-outward sequence. The outermost arrows indicate the positions and transcriptional orientations of the open reading frames; Figure S3: The proportion of *bla*_NDM_ variants across different species and the network graph depicting the coexistence patterns of different *bla*_NDM_ gene variants with other ARGs, ISs and plasmid replicons harbored in different bacteria. (A) The bar chart shows the percentage of *bla*_NDM_ variants in *bla*_NDM_-positive strains of different species. (B,C) The network graph illustrates the correlations between *bla*_NDM_ variants and other genetic elements in *bla*_NDM_-positive strains of different species. The nodes represent ARGs, ISs and plasmid replicons identified in all *bla*_NDM_-positive strains of from different species. The connections between nodes signify their interrelatedness. Blue hues and increased line thickness denote stronger positive correlations. The intensity of the yellow color on the lines indicates the strength of negative correlations, with darker shades of yellow corresponding to stronger negative correlations. Additionally, the thickness of the lines is directly proportional to the correlation strength, where a thicker line signifies a more pronounced relationship between the variables. All associated genes depicted in the figure exhibited *p* values less than 0.05; Figure S4: The network graph depicting the coexistence patterns of different *bla*_NDM_ gene variants with other ARGs, ISs and plasmid replicons harbored in different bacteria. (A–C). The network graph illustrates the correlations between *bla*_NDM_ variants and other genetic elements in *bla*_NDM_-positive strains of different species. The nodes represent ARGs, ISs and plasmid replicons identified in all *bla*_NDM_-positive strains of from different species. The connections between nodes signify their interrelatedness. Blue hues and increased line thickness denote stronger positive correlations. The intensity of the yellow color on the lines indicates the strength of negative correlations, with darker shades of yellow corresponding to stronger negative correlations. Additionally, the thickness of the lines is directly proportional to the correlation strength, where a thicker line signifies a more pronounced relationship between the variables. All associated genes depicted in the figure exhibited *p* values less than 0.05.
