# Supplementary material for: Genomic Insights into Carbapenem-Resistant Organisms Producing New Delhi Metallo-β-Lactamase in Live Poultry Markets
Source: Microorganisms. 2025 May 23;13(6):1195. doi: 10.3390/microorganisms13061195 (PMC12194966; doi:10.3390/microorganisms13061195)
Supplement: Supplementary file 1 [file microorganisms-13-01195-s001.zip › Supplementary figures.pdf]

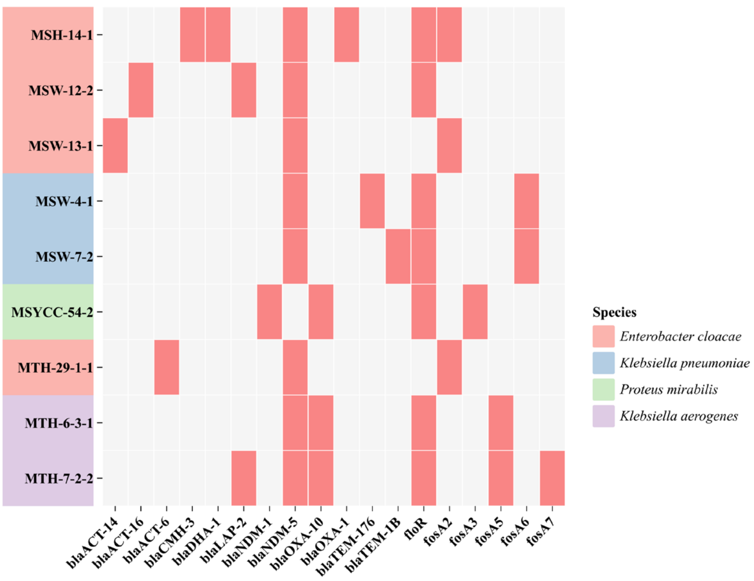

Figure S1. The prevalence of ARGs harbored by *E. cloacae*, *K. aerogenes*, *K. pneumoniae* and *P. mirabilis*.

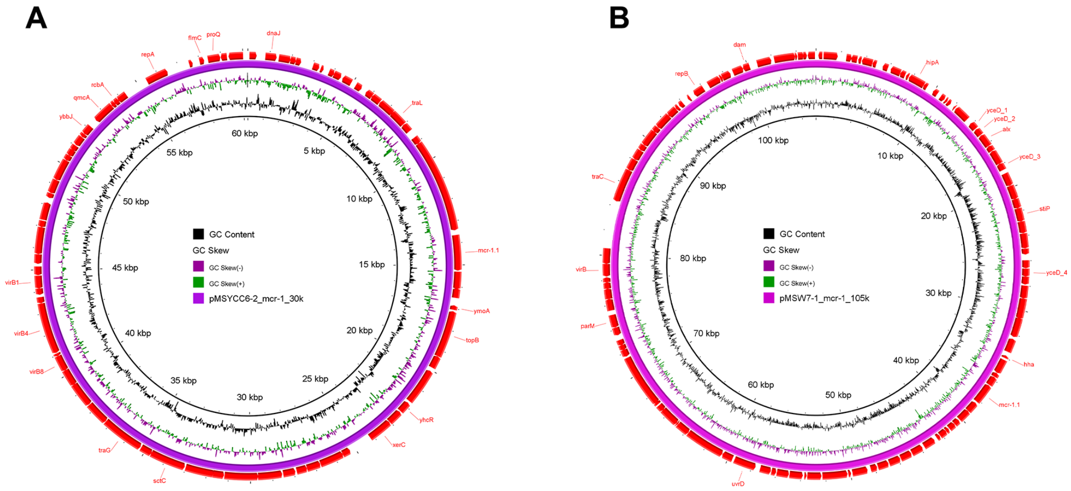

Figure S2. Plasmid profiles of two *mcr-1*-containing plasmids. The annotation of two plasmid slices (assembled contigs, not complete plasmids) from this study. The GC skew and GC content are depicted in an inward-to-outward sequence. The outermost arrows indicate the positions and transcriptional orientations of the open reading frames.

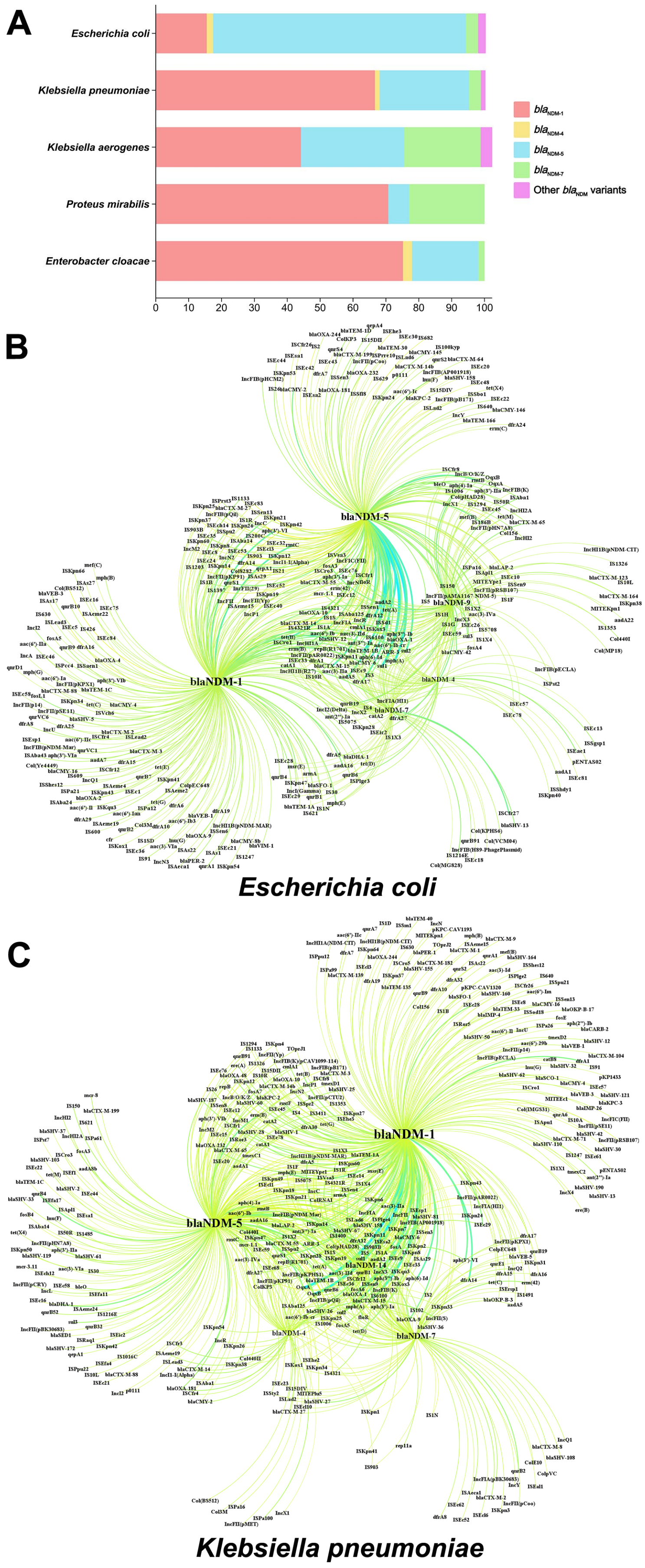
A
Escherichia coli
Klebsiella pneumoniae
Klebsiella aerogenes
Proteus mirabilis
Enterobacter cloacae
0
10
20
30
40
50
60
70
80
90
100
blaNDM-1
blaNDM-4
blaNDM-5
blaNDM-7
Other blaNDM variants
B
blaNDM-5
blaNDM-9
blaNDM-1
blaNDM-4
blaNDM-7
Escherichia coli
C
blaNDM-1
blaNDM-5
blaNDM-14
blaNDM-4
blaNDM-7
Klebsiella pneumoniae

Figure S3. The proportion of *bla*$_{NDM}$ variants across different species and the network graph depicting the coexistence patterns of different *bla*$_{NDM}$ gene variants with other ARGs, ISs and plasmid replicons harbored in different bacteria. (A) The bar chart shows the percentage of *bla*$_{NDM}$ variants in *bla*$_{NDM}$-positive strains of different species. (B-C) The network graph illustrates the correlations between *bla*$_{NDM}$ variants and other genetic elements in *bla*$_{NDM}$-positive strains of different species. The nodes represent ARGs, ISs and plasmid replicons identified in all *bla*$_{NDM}$-positive strains of from different species. The connections between nodes signify their interrelatedness. Blue hues and increased line thickness denote stronger positive correlations. The intensity of the yellow color on the lines indicates the strength of negative correlations, with darker shades of yellow corresponding to stronger negative correlations. Additionally, the thickness of the lines is directly proportional to the correlation strength, where a thicker line signifies a more pronounced relationship between the variables. All associated genes depicted in the figure exhibited p values less than 0.05.

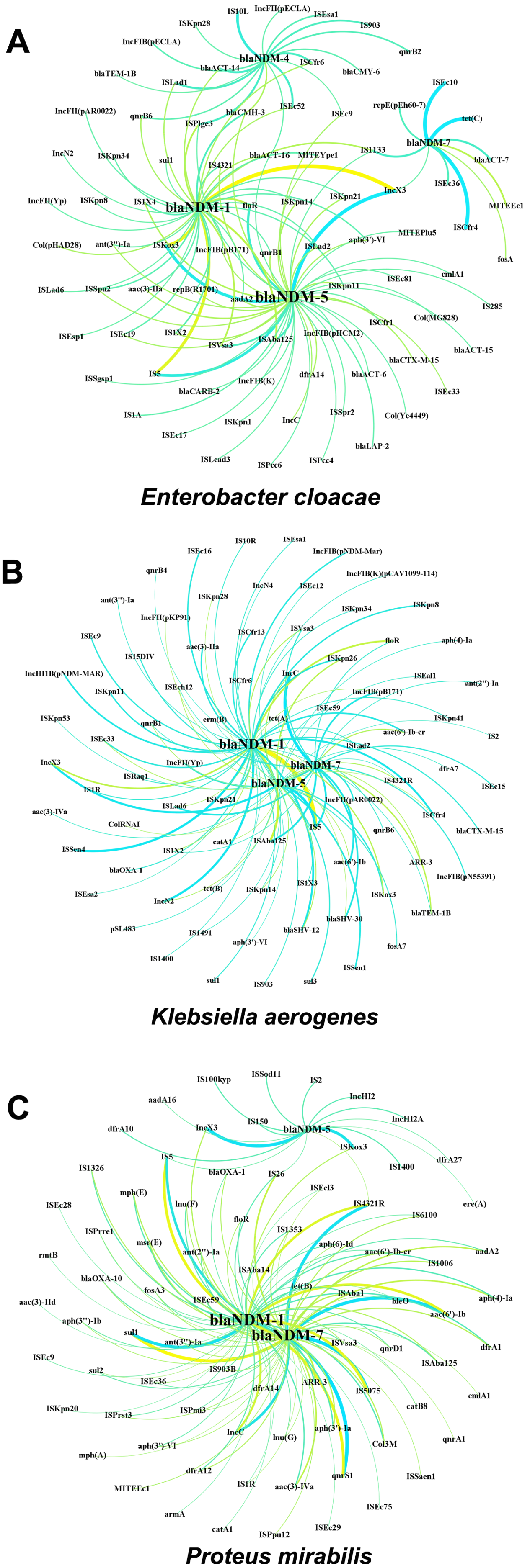
A
IS10L
IncFII(pECLA)
ISEsa1
IS903
qnrB2
ISKpn28
IncFIB(pECLA)
blaNDM-4
ISCfr6
blaACT-14
blaCMY-6
blaTEM-1B
ISLad1
ISEc10
repE(pEh60-7)
ISEc52
ISEc9
tet(C)
IncFII(pAR0022)
qnrB6
blaCMH-3
ISPlge3
blaNDM-7
IncN2
ISKpn34
sul1
IS4321
blaACT-16
MITEYpe1
IS1133
blaACT-7
ISEc36
IncX3
MITEEc1
IncFII(Yp)
ISKpn8
IS1X4
blaNDM-1
floR
ISKpn14
ISKpn21
ISCfr4
MITEPlu5
Col(pHAD28)
ant(3'')-Ia
ISKox3
IncFIB(pB171)
qnrB1
ISLad2
aph(3')-VI
fosA
ISLad6
ISSpu2
aac(3)-IIa
repB(R1701)
aadA2
blaNDM-5
ISKpn11
ISEc81
cmlA1
ISEsp1
ISEc19
IS1X2
ISVsa3
ISAba125
IncFIB(pHCM2)
ISCfr1
Col(MG828)
IS285
blaACT-15
blaCTX-M-15
ISSgsp1
IS5
IncFIB(K)
dfrA14
blaACT-6
ISEc33
blaCARB-2
IS1A
ISKpn1
IncC
ISSpr2
Col(Ye4449)
ISEc17
ISLead3
ISPcc6
ISPcc4
blaLAP-2
Enterobacter cloacae
B
IS10R
ISEsa1
ISEc16
IncFIB(pNDM-Mar)
qnrB4
IncFIB(K)(pCAV1099-114)
IncN4
ISEc12
ant(3'')-Ia
ISKpn28
ISKpn34
ISKpn8
IncFII(pKP91)
ISCfr13
ISVsa3
floR
aph(4)-Ia
ISEc9
aac(3)-IIa
ISKpn26
IS15DIV
IncHI1B(pNDM-MAR)
ISCfr6
IncC
ISEal1
ant(2'')-Ia
ISKpn11
ISEch12
IncFIB(pB171)
ISKpn53
qnrB1
erm(B)
tet(A)
ISEc59
ISKpn41
ISEc33
blaNDM-1
aac(6')-Ib-cr
IS2
ISLad2
IncX3
IncFII(Yp)
blaNDM-7
dfrA7
ISRaq1
blaNDM-5
IS4321R
ISEc15
IS1R
ISKpn21
IncFII(pAR0022)
aac(3)-IVa
ISLad6
ISCfr4
ColRNAI
IS5
blaCTX-M-15
catA1
ISAba125
qnrB6
ISSen4
IS1X2
aac(6')-Ib
ARR-3
blaOXA-1
IncFIB(pN55391)
ISEsa2
tet(B)
IS1X3
ISKox3
IncN2
ISKpn14
blaTEM-1B
pSL483
IS1491
blaSHV-30
blaSHV-12
aph(3')-VI
fosA7
IS1400
ISSen1
sul1
IS903
sul3
Klebsiella aerogenes
C
IS100kyp
ISSod11
IS2
aadA16
IncHI2
IS150
IncHI2A
dfrA10
IncX3
blaNDM-5
ISKox3
IS5
IS1400
dfrA27
IS1326
blaOXA-1
IS26
ISEcl3
ere(A)
ISEc28
mph(E)
lnu(F)
IS4321R
floR
IS6100
ISPrre1
IS1353
msr(E)
aph(6)-Id
aadA2
rmtB
ant(2'')-Ia
aac(6')-Ib-cr
IS1006
blaOXA-10
ISAba14
tet(B)
fosA3
ISAba1
aac(3)-IId
ISEc59
bleO
aph(4)-Ia
aph(3'')-Ib
blaNDM-1
aac(6')-Ib
sul1
blaNDM-7
ant(3'')-Ia
ISVsa3
dfrA1
qnrD1
ISEc9
IS903B
ISAba125
sul2
ARR-3
ISEc36
IS5075
dfrA14
cmlA1
ISKpn20
ISPrst3
ISPmi3
catB8
aph(3')-Ia
IncC
lnu(G)
Col3M
qnrA1
mph(A)
aph(3')-VI
dfrA12
qnrS1
ISSaen1
IS1R
aac(3)-IVa
MITEEc1
ISEc75
armA
catA1
ISPpu12
ISEc29
Proteus mirabilis

Figure S4. The network graph depicting the coexistence patterns of different *bla*$_{\text{NDM}}$ gene variants with other ARGs, ISs and plasmid replicons harbored in different bacteria. (A-C) The network graph illustrates the correlations between *bla*$_{\text{NDM}}$ variants and other genetic elements in *bla*$_{\text{NDM}}$-positive strains of different species. The nodes represent ARGs, ISs and plasmid replicons identified in all *bla*$_{\text{NDM}}$-positive strains of from different species. The connections between nodes signify their interrelatedness. Blue hues and increased line thickness denote stronger positive correlations. The intensity of the yellow color on the lines indicates the strength of negative correlations, with darker shades of yellow corresponding to stronger negative correlations. Additionally, the thickness of the lines is directly proportional to the correlation strength, where a thicker line signifies a more pronounced relationship between the variables. All associated genes depicted in the figure exhibited p values less than 0.05.
